# Transcription factor Foxp1 stimulates angiogenesis in adult rats after myocardial infarction

**DOI:** 10.1038/s41420-022-01180-5

**Published:** 2022-09-10

**Authors:** Dinghui Wang, Bin Liu, Tianhua Xiong, Wenlong Yu, Huiping Yang, Jing Wang, Xiaodong Jing, Qiang She

**Affiliations:** Department of Cardiology, The Second Affiliated Hospital of Chongqing Medical University, Chongqing, 400010 China

**Keywords:** Biological metamorphosis, Collective cell migration

## Abstract

Forkhead box protein P1 (FoxP1) is essential for cardiac development and the regulation of neovascularization, but its potential for cardiac angiogenesis has not been explored. This study aims to investigate the angiogenic role of FoxP1 in a rat model of myocardial infarction (MI). Adult male rats were subjected to MI, and *Foxp1* was knocked down with lentivirus FoxP1 siRNA. Endothelial cell proliferation, angiogenesis, and cardiac function were also assessed. Cell scratch assay and tubule formation analysis were used to detect the migration ability and tube formation ability of human umbilical vein endothelial cells (HUVECs). Compared with that in the sham group, results showed that the expression of FoxP1 was significantly increased in the MI group. *Foxp1* knockdown decreases FoxP1 expression, reduces angiogenesis, and increases collagen deposition. When *Foxp1* was knocked down in HUVECs using FoxP1 siRNA lentivirus, cell proliferation, migration, and tube formation abilities decreased significantly. Our study showed that FoxP1 elicits pleiotropic beneficial actions on angiogenesis in the post-MI heart by promoting the proliferation of endothelial cells. FoxP1 should be considered a candidate for therapeutic cardiac angiogenesis.

## Introduction

Coronary heart disease is one of the dominant causes of death worldwide and poses a serious economic burden [1, 2]. Ischemia and hypoxia caused by sudden occlusion of the main coronary artery lead to myocardial infarction (MI) [1, 3]. Although advancements in the current treatment of coronary heart disease and timely revascularization through emergency percutaneous coronary intervention in most patients with acute MI have accomplished the removal of the blocked coronary artery [4], the long-term mortality remains high. In addition, sequelae and side effects, such as stent thrombosis or stent restenosis and gastrointestinal or oral bleeding caused by the long-term use of antiplatelet drugs, hinder the prognosis of MI. Therefore, therapeutic strategies that facilitate the recovery of the non-functioning microvasculature and treatment options are necessary to treat patients with MI. In recent years, myocardial revascularization has become a research focus in the field of MI treatment, and with the rapid development of molecular biology technology, remedial angiogenesis has become a promising new treatment for patients with MI [5, 6]. Researchers believe that wound healing after MI is related to a strong angiogenic response that starts in the marginal zone and extends to the necrotic infarct core [7]. In a study on heart tissue regeneration and protection, Slater and colleagues found that insulin-like growth factor-1 could inhibit the apoptosis of cardiomyocytes, recruit endogenous stem cells, and promote angiogenesis [8]. The fibroblast growth factor family and vascular endothelial growth factor family have been used in animal experiments and in patients with MI; however, their neoangiogenic capability is not viable for cardiac generation [9, 10].

The forkhead box (Fox) family comprises 17 subfamilies with more than 100 members, and its DNA-binding region has a special wing-like spiral structure [11]. The proteins encoded by the Fox family have diverse functions, and are not only involved in glucose and lipid metabolism, species aging, and immune regulation processes, but are also related to embryonic development and cell cycle regulation [12]. FoxP1 is a member of the P subfamily of the Fox transcription factor superfamily and is mainly expressed in overlapping patterns in lung, nerve, lymph, and heart tissues [13, 14]. Studies have reported that FoxP1 is found in the entire heart, including the myocardium, endocardium, and endocardial cushion tissue at E11.5, and FoxP1 was also discovered in aortic and pulmonary artery endothelial cells at E12.5d and E14.5d [15]. In the ApoE knockout (KO) hyperlipidemic mouse model, the loss of FoxP1 in endothelial cells promotes atherosclerosis, and endothelial FoxP1 affects the adhesion, migration, and penetration of monocytes into the vascular wall in the pathogenesis and progression of atherosclerosis [16]. Wang et al. observed that FoxP1 KO mice died at the embryonic stage at E14.5, due to experiencing perivascular hemorrhage and edema [17]. In a study on angiogenesis, it was found that the expression of *Foxp1* was upregulated at the neovascular site after ischemic induction in the hind limbs of mice [18]. At present, studies on FoxP1 in cardiac function have mainly focused on myocardial cell proliferation and atherosclerosis. It has been reported that FoxP1 can produce a marked effect in inhibiting myocardial fibrosis and cardiac remodeling. However, a relationship between FoxP1 and angiogenesis after MI remains unreported. Our data indicates that FoxP1 most likely plays a pro-angiogenic role by promoting endothelial cell proliferation in the post-MI heart. We also intend to clarify the mechanism involved in this process.

## Materials and Methods

### Experimental animals

Male Sprague‒Dawley rats were obtained from the Experimental Animal Center of Chongqing Medical University. All the animals in this study received humane care in compliance with the “Guide for the Care and Use of Laboratory Animals”. All animal experiments were approved by the Institutional Animal Care and Use Committee of Chongqing Medical University.

### MI model

MI was induced by ligation of the left coronary artery in rats. The animal sample size was estimated by the degree of freedom (E) of analysis of variance. E is equal to sum of experimental animals in each group minus number of groups. Thirty male rats, aged 8–12 weeks, were completely randomized into three groups using random number table, then underwent either coronary artery ligation or sham surgery (thoracotomy without coronary ligation). Anesthesia was induced intraperitoneally using pentobarbital sodium (30–50 mg/kg). A left-sided thoracotomy was performed, and the left anterior descending artery branch was exposed and ligated approximately 4 mm from its origin. Subsequently, positive pressure was applied to the lungs by a ventilator, and then the chest wall was closed. The elevation of the ST segment on the electrocardiogram (ECG) was a sign of successful MI surgery. Echocardiography was performed four weeks after operations.

### Cell culture

Human umbilical vein endothelial cells (HUVECs) were purchased from the company (ATCC, Rockville, MD, USA), cultured in RPMI 1640 medium supplemented with penicillin, streptomycin, and 10% fetal bovine serum, and used for the 10^th^ generation. Stable-growing HUVECs were seeded in a six-well plate at a density of 2 × 10^4^ cells/well and placed in a cell incubator overnight. The cells were removed the next day and placed in a hypoxic cell incubator (5% O_2_) for different durations.

### Lentiviral transfection

The pre-experiment result showed that the most suitable multiplicity of infection was 10, and the formal lentiviral transfection experiment was conducted according to the manufacturer’s instructions. HUVECs were seeded in six-well plates at a density of 3–5 × 10^4^ cells/well. Complete medium, 1 × 10^8^ TU/mL negative control virus, and 1 × 10^8^ TU/mL FoxP1 interfering RNA lentivirus were added to the control group, vehicle group, and FoxP1 RNAi group HUVECs, respectively. The infection volume was 2 mL and the recommended optimal conditions were used for infection conditions. The fluid was changed the day after infection. Three days after infection, lentiviral infection efficiency and cell status were observed using a fluorescence microscope.

### Western blot analysis

For western blot analysis of FoxP1 expression, cultured cells were lysed in RIPA buffer with protease inhibitor phenylmethylsulfonyl fluoride (PMSF) on ice, and the protein concentration was quantified according to the recommended protocol. Equal amounts of total protein (15 μg) were separated by 10% sodium dodecyl sulfate-polyacrylamide gel electrophoresis (SDS-PAGE) and transferred to polyvinylidene fluoride (PVDF) membranes (Millipore, Billerica, MA, USA). The membrane was blocked with 5% milk in Tris-buffered saline Tween (TBST) (50 mM Tris, 150 mM NaCl, 0.5 mM Tween-20, pH = 7.5), and then were incubated overnight with antibodies directed against human FoxP1. β-actin was used as the loading control. The membranes were then washed carefully and incubated with a secondary antibody. Finally, the immunoblots were visualized using enhanced chemiluminescence.

### Immunofluorescence

Rat hearts were acquired and fixed with 4% polyformaldehyde, dehydrated, embedded in paraffin, and cut into 4-μm-thick sections. Fixed cells or tissue sections were permeabilized with 0.1% Triton X-100 in PBS (0.01 mol/L) at 37 °C for 10 min. Cells or sections were blocked with 10% goat serum (Boster Biological Technology, Pleasanton, CA, USA) at 37 °C for 10 min and then incubated with the following primary antibodies at 4 °C overnight (Anti-FOXP1 antibody (abcam,ab16645),anti-CD31 antibody (abcam,ab282746), anti-Ki67 antibody (CST, 9129), anti-P-Histone H3 antibody (CST, 53348)). The cells were then incubated with secondary antibodies (Tetramethylrhodamine (TRITC)-conjugated goat anti-mouse IgG (abcam, ab150115), fluorescein-conjugated goat anti-rabbit IgG (abcam, ab150077) at 37 °C for 45 min, followed by incubation with DAPI at room temperature for 10 min. Subsequently, the cells were subjected to confocal microscopy (original magnification, ×400). The imaging conditions for each antibody were consistent across all samples.

### RNA extraction and quantitative real-time PCR (RT‑qPCR)

Cells were collected and lysed with TRIzol (Takara Bio Inc., Otsu, Japan) to extract total RNA, which was reverse transcribed to complementary DNA using a PrimeScript Reverse Transcriptase reagent kit (cat. no. RR047A; Takara Bio Inc.), according to the manufacturer’s protocol. RT-qPCR for β-actin and FoxP1 was performed. Bio-Rad software (version 9.5) was used to analyze the qPCR results, including melting curves, and utilized a comparative cycle threshold to count relative mRNA expression levels. The experiments were repeated three times, and the mRNA expression was normalized to that of β-actin. Primer sequences used are listed below: FoxP1: forward: GCAGTGTGCGAAGATTTCCAA, reverse: TCACATGCAGGTGGGTCATC. β-actin: forward: GGCTGTATTCCCCTCCATCG; reverse: CCAGTTGGTAACAATGCCATGT.

### Flow Cytometry

HUVECs were digested with 0.25% trypsin to obtain single-cell suspensions. Next, the cells were centrifuged at 300 times the gravity for 5 min and resuspended in 0.1 mL phosphate buffered solution. Subsequently, the cells were fixed by slowly adding 500 mL chilled 75% alcohol. Then, 50 mg/mL propidium iodide was used to stain the cells for 30 min in a dark. Finally, cell cycle and apoptosis were analyzed using Cell Quest software (BD Biosciences, Franklin Lakes, NJ).

### Masson Staining

The rat heart tissue sections were stained with Masson’s trichrome stain kit (G1340-7, Solarbio). After routine dewaxing in water, the sections were stained with Weigert’s hematoxylin for 5 min, washed again with tap water for 5 min, and rinsed in distilled water. Next, the slides were stained with fuchsin, rinsed in distilled water, incubated in phosphotungstic‒phosphomolybdic acid for 5 min, stained with aniline blue for 5 min, and fixed in 1% acetic acid for 2 min. Finally, the slides were rinsed with distilled water, dehydrated, and mounted.

### Scratch test

Before the experiment, six horizontal lines were drawn on the back of the six-well plate with an interval of about 0.5 to 1 cm to make the lines evenly pass through the holes. Approximately 5 × 10^5^ HUVECs were added to the six-well plate and placed in an incubator for overnight culture. The next day, a liner wound was made using a 200 uL pipette tip and a ruler perpendicular to the horizontal line. The cells were washed 3 times with sterile PBS buffer and 2 mL of serum-free simple medium was added to each well. The 5% CO_2_ incubator at 37 °C was used to continue cultivation. The size of the wounds was observed, and images were taken under an inverted phase-contrast microscope at 0, 6, 12, and 24 h after the scratch.

### Tubule formation experiment

The pre-cooled Matrigel Matrix (BD Biosciences, USA) was spread in a 96-well plate at 50 μL/well, placed in a 5% CO_2_ incubator at 37 °C for 60 min for its polymerization; 100 µL HUVECs suspension was seeded on the surface of each well at a density of 1.5 × 10^5^ cells/mL and cultured for 2 h. Next, the number of cells in each group was observed under an inverted phase-contrast microscope. Obvious tubular structures were visible in 2.5 h; six fields of view were randomly selected for image capturing, and then ImageJ (Version: 1.8.0) software was used for the analysis and processing.

### Statistical analysis

Statistical analyses were performed using SPSS version 27.0 software (SPSS, Chicago, IL, USA). Data were presented as mean ± standard deviation (SD) or median (interquartile range, IQR). Comparisons between 2 groups were analyzed using Student’s t test. Differences among continuous variables were estimated using one-way ANOVA and the Kruskal‒Wallis H test. For each test, a two-tailed *p* value below 0.05 was defined as statistically significant.

## Results

### The expression of FoxP1 increased in the heart after MI in rats

**Fig. 1:**
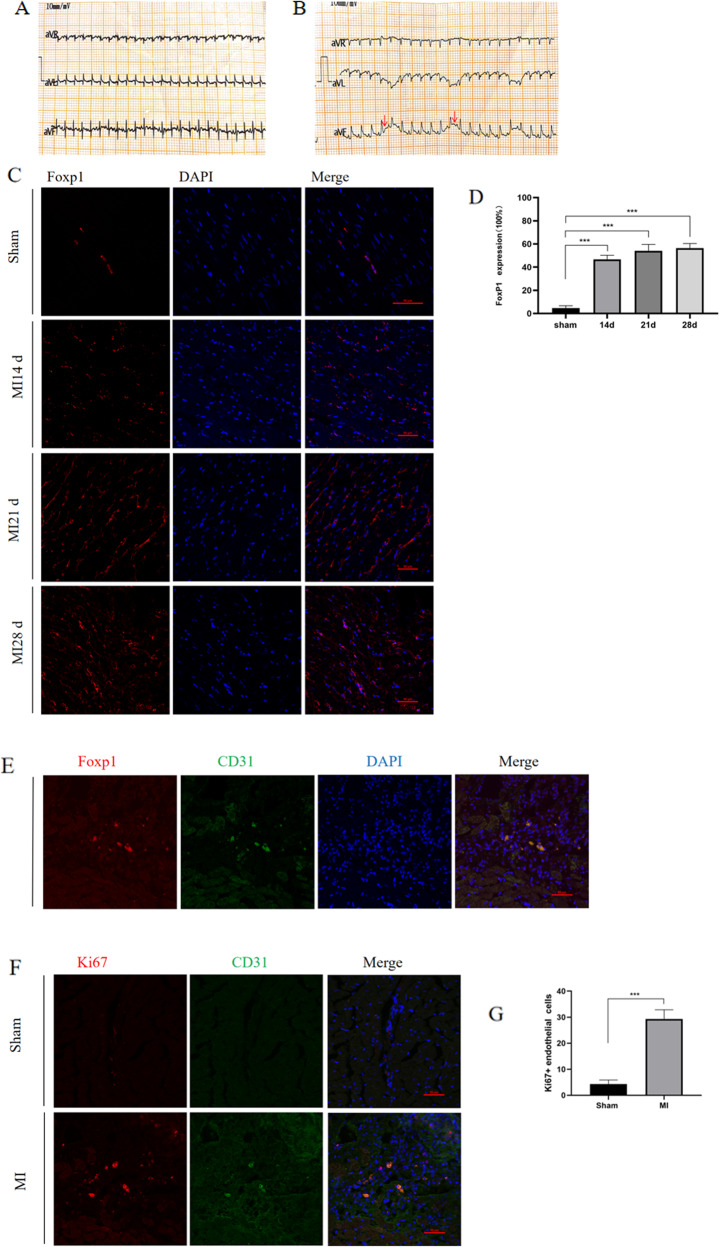
The expression of FoxP1 protein in the heart after myocardial infarction in rats. **A**, **B** Electrocardiogram of limb leads before and after myocardial infarction in rats. **C**, **D** FoxP1 expression in heart tissue of sham-operated rats and 14 days 21 days and 28 days after MI. (*N* = 3) **E** endothelial markers CD31 and FoxP1 and nuclear staining (DAPI) immunofluorescence double staining localization showed FoxP1 expression in the vascular endothelial cells of the heart after MI. **F**, **G** Endothelial cell proliferation in myocardial infarction group and sham-operated group was detected by confocal fluorescence of endothelial cell-specific marker CD31 and proliferation index Ki67 (*N* = 3). The data were approximately normal distribution and the variance is homogeneous, One-way ANOVA with post hoc LSD multiple comparison test (**D**) and two independent samples Student’s t test (**G**) were used. ***P < 0.001, Scale bar = 50 mm in **C**, **E** and **F**.

To explore the role of FoxP1 in cardiovascular angiogenesis in adult rats, we generated an MI model, as described previously. After the operation, ECG of the limb lead monitoring showed an obvious ST-segment elevation (Fig. 1A, B). Subsequently, we observed that the expression of *Foxp1* increased significantly in rats’ post-MI heart at 14, 21, and 28 days, compared with that in the sham operation group (Fig. 1C, D). Interestingly, we found that there was no significant difference in *Foxp1* expression among MI group at 14, 21, and 28 days. As shown in Fig. 1E, results illustrated that FoxP1 is expressed in capillary endothelial cells and *Foxp1* is upregulated at sites of neovascularization. Additionally, results showed that fluorescence of Ki67 in the MI group was stronger than that in the sham group (Fig. 1F, G). The above results indicate that endothelial cell proliferation in the post-MI heart, the most crucial part of angiogenesis, may be regulated by the transcription factor FoxP1.

### Foxp1 knockdown affects angiogenesis and cardiac function in MI rats

**Fig. 2:**
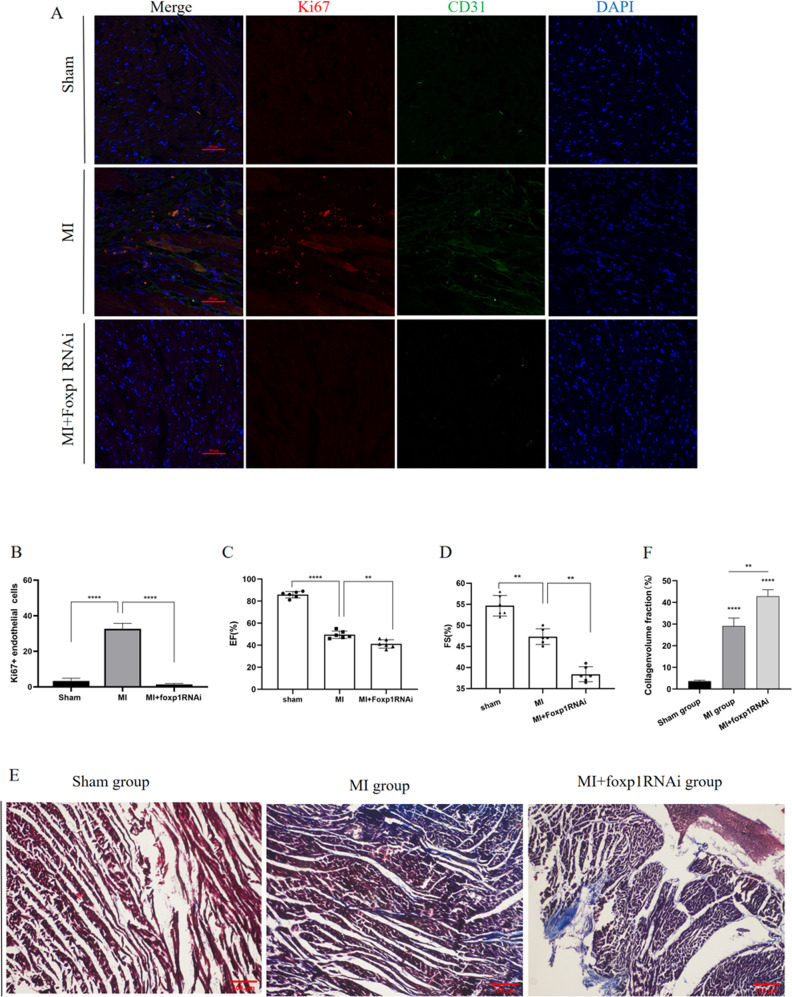
Foxp1 knockdown affects angiogenesis and cardiac function in MI rats. **A**, **B** ompared with MI group, endothelial cells proliferated less in the MI + Foxp1 RNAi group. (*N* = 6) **C**, **D** Cardiac function of three groups of rats. **E**, **F** Masson staining of rat heart tissue of three groups, results showed that fibrosis was aggravated while foxp1 knockdown, and blue indicated collagen deposition (*N* = 6). The data were approximately normal distribution and the variance is homogeneous, One-way ANOVA with post hoc LSD multiple comparison test was used (**B**, **C**, **D**, **F**). **P < 0.01,***P < 0.001, ****P < 0.0001, scale bar = 100 mm in **A** and **D**.

To further investigate whether FoxP1 is a factor in the regulation of angiogenesis after myocardial ischemia, a heart-specific FoxP1 knockdown model was constructed using the dot matrix injection method to inject FoxP1 interfering RNA lentivirus into the hearts of MI rats. Four weeks later, color Doppler echocardiography was performed to detect cardiac function. As shown in Fig. 2A, B, *Foxp1* knockdown of endothelial cell proliferation was clearly lower than that in the MI group, suggesting that FoxP1 knockdown does affect cardiac angiogenesis in rats with MI. Echocardiography results showed that the left ventricular ejection fraction of the FoxP1 knockdown group was lower than that of the myocardial infarction group (Fig. 2C, D). The results revealed that the MI group had significantly more collagen deposition than the sham operation group according to the collagen volume fraction, and the myocardial tissue was more disordered; however, FoxP1 knockdown led to more severe collagen deposition and more obvious fibrosis (Fig. 2E, F). These results suggested that *Foxp1* knockdown reduced cardiac angiogenesis and cardiac function, and worsened fibrosis in rats with myocardial infarction.

### *Foxp1* knockdown affects endothelial cell proliferation

**Fig. 3:**
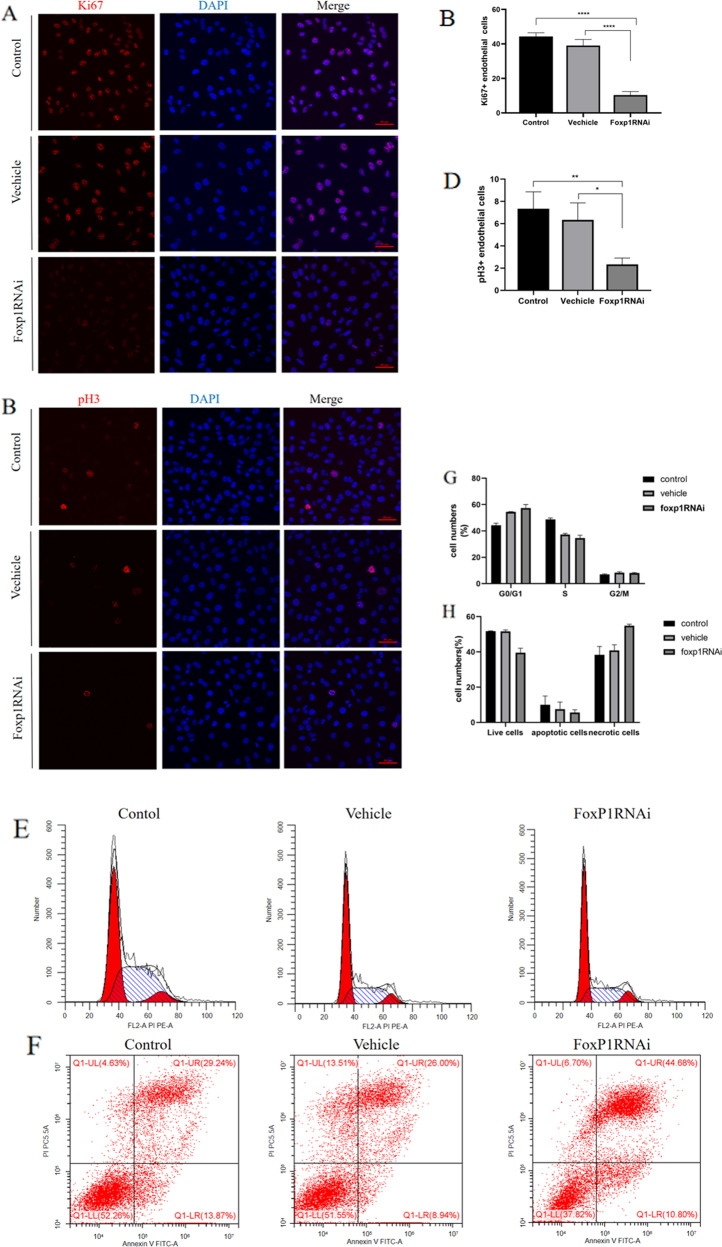
The proliferation of HUVECs in the FoxP1RNAi group were obviously reduced compared with control and vehicle group (ie, the negative virus group). **A**–**D** Quantification of the fluorescent intensity of Ki67 and pH3 immunostaining in HUVEC of control group, vehicle group and FoxP1RNAi group (*N* = 6). **E**, **G** The proportion of cells in G1/S/G2 phase of control, vehicle and FoxP1RNAi group was determined by flow cytometry (*N* = 6). **F**, **H** Flow cytometry detection of HUVEC cell apoptosis in the control group, vehicle group and FoxP1RNAi group. (*N* = 3). The data were approximately normal distribution and the variance is homogeneous, One-way ANOVA with post hoc LSD multiple comparison test was used (**B**, **D**, **G**, **H**). *P < 0.05, **P < 0.01,***P < 0.001, ****P < 0.0001, scale bar = 50 mm in **A** and **B**.

Similarly, the interfering RNA lentivirus of FoxP1 was transfected into HUVECs according to the instructions (Fig. S1). As shown in Fig. 3A‒D, after knocking down the *Foxp1* gene, endothelial cells were prominently reduced compared with the control and vehicle groups by detecting the number of the proliferation index Ki67 and PH3 positive cells. The results of flow cytometry also showed that the HUVECs with double to quadruple DNA, that is, cells in the G1‒S phase and the S‒G2 phase in the FoxP1 knockdown group, were significantly less than those in the control and vehicle groups (Fig. 3E, G). As expected, compared to that in the control group, the number of apoptotic HUVECs in the FoxP1 knockdown group was significantly increased (Fig. 3F, H). We hypothesized that this was due to knockdown of *Foxp1* that endothelial cell proliferation was attenuated, indicating that FoxP1 plays an indispensable role in endothelial cell proliferation.

### Endothelial cell migration reduced by knocking down *Foxp1*

**Fig. 4:**
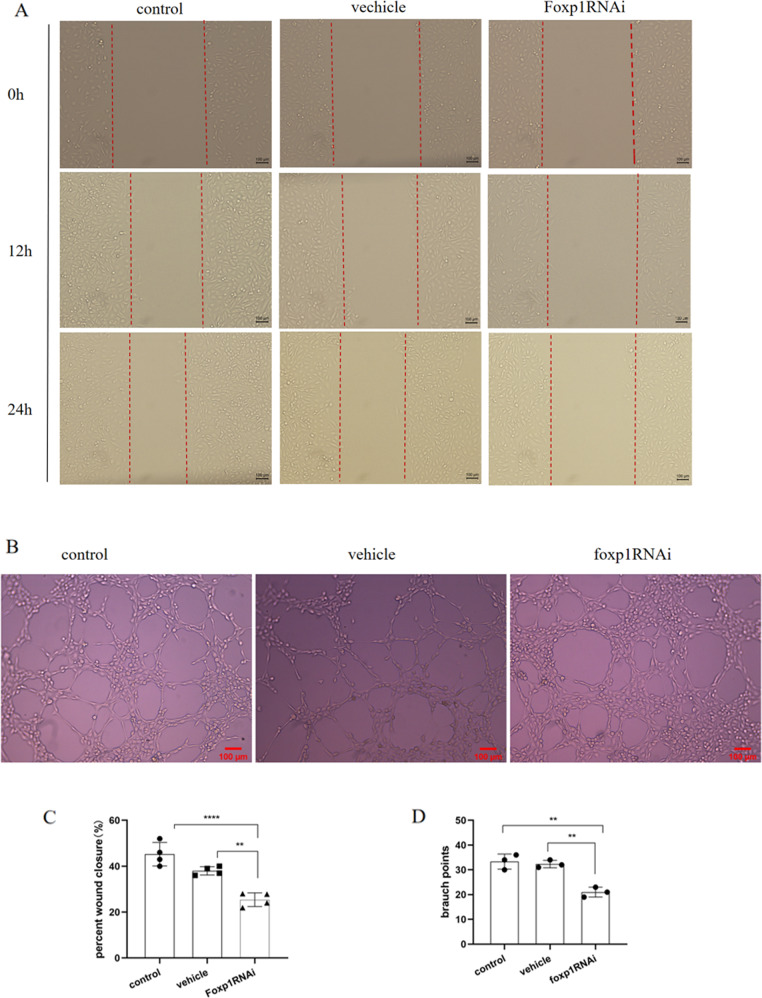
The migration ability of endothelial cells with knockdown of FoxP1 gene was strongly decreased compared with the normal control and negative control virus groups. **A**, **C** Scratch test results of HUVEC cells in each group, after 24 hours of scratching, the number of cells migrated in the experimental group was rapidly reduced by half compared with the control group (*N* = 4). **B**, **D** Three groups of HUVEC cell tubule formation experiment results. (*N* = 3). The data were approximately normal distribution and the variance is homogeneous, One-way ANOVA with posthoc LSD multiple comparison test was used (**C**, **D**). **P < 0.01, ****P < 0.0001. Scale bar = 100 mm in **A** and **B**.

It is well known that angiogenesis is accompanied by the formation and migration of endothelial cell migration tubules in addition to the proliferation of endothelial cells. Therefore, a scratch test was performed to examine the migratory ability of HUVECs under different conditions. The migratory ability of endothelial cells with knockdown of *Foxp1* was strongly decreased compared to that of the control and vehicle groups (Fig. 4A, C). In addition, we performed a tubule formation assay using Matrigel, as shown in Fig. 4B, D, and the tubules formed in the three groups were remarkably distinct. When the *Foxp1* gene was knocked down, the tubule formation was less and the shape was more irregular, with the majority of the cells disordered or damaged. Thus, it can be inferred that FoxP1 has a positive effect in promoting endothelial cell migration and the formation of new blood vessels.

### Hypoxic culture of endothelial cells induces increased FoxP1 expression

**Fig. 5:**
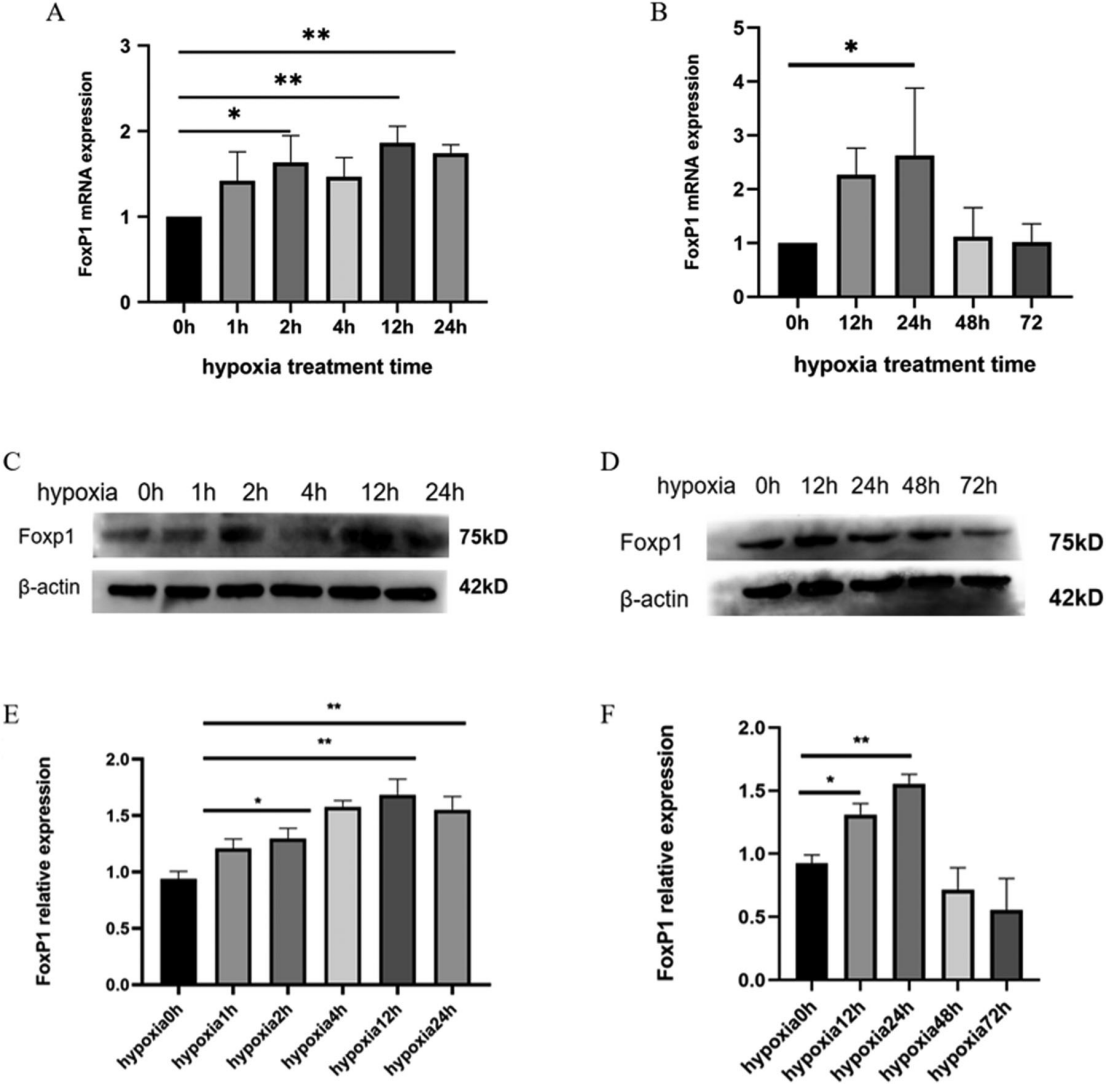
Expression of foxp1 in endothelial cells cultured under hypoxia. **A**, **B** Foxp1 expression by qPCR in HUVECs after hypoxic treating HUVEC for a short time and for a long time (*N* = 3). **C**–**F** Changes in FoxP1 protein expression after hypoxic treating HUVEC for a short time and for a long time (*N* = 3). The data were approximately normal distribution and the variance is homogeneous, One-way ANOVA with post hoc LSD multiple comparison test was used (**A**, **B**, **E**, **F**). *P < 0.05,**P < 0.01.

To narrow down the relationship between angiogenesis and FoxP1, we established a short-term hypoxic group and a long-term hypoxic group of well-growing HUVECs, as HUVECs are one of the most used cells for the study of angiogenesis. The short-term hypoxic group includes 0, 1, 2, 4, 12, and 24 h, and the long-term hypoxic group contains 0, 12, 24, 48, and 72 h. The results showed that within 24 h of short-term hypoxia, the expression of FoxP1 was significantly increased compared with that in the control group (*p* < 0.05) (Fig. 5A). However, after 24 h, the expression of FoxP1 decreased and was lower than that in the control group (Fig. 5B). The FoxP1 protein also increased within 24 h of hypoxic culture, which gradually decreased at 48 and 72 h (Fig. 5C‒F). The data indicates that the hypoxic microenvironment affects the expression of FoxP1, and proper hypoxic cultivation of endothelial cells increases the expression of FoxP1.

## Discussion

Angiogenesis refers to the process by which vascular endothelial cells proliferate, migrate, and form new microvessels in the existing vasculature under pathological and physiological conditions, such as injury repair and tissue ischemia regeneration [19]. Angiogenesis also has the potential to treat and improve tissue perfusion in ischemia, and insufficient angiogenesis can lead to coronary artery disease. Capillary injury creates a hypoxic environment in wounds, and experimental changes in wound oxygenation alter the recovery of angiogenic responses. Based on the molecular characterization of several angiogenic growth factors, an early study showed that hypoxia can induce mRNA expression of platelet-derived growth factor and vascular endothelial growth factor in tissue culture [20].

Fox family of proteins regulate gene transcription and control cell- and tissue-specific gene expression during early development and organogenesis [21]. Among Fox transcription factors, members of the Fox P subfamily (FoxP1, FoxP2, and FoxP3) have recently attracted attention. The Fox family has diverse functions in various physiological and pathophysiological processes, ranging from speech development (FoxP2) [22] to immune regulatory T cells (FoxP3) [23] and cardiogenesis (FoxP1, FoxP4) [24]. FoxP1 was first mentioned in the cardiovascular field by Wang et al., who described the phenotype of hereditary FoxP1 deficiency in embryonic stem cells of mice [15]: Inactivation of FoxP1 leads to severe cardiac defects, including poor separation of the ventricle and outflow tract and valve formation. In cardiac tissue, FoxP1 is expressed in both the myocardium and endocardium and is involved in the transcriptional regulation of different types of cells [25, 26], such as cardiomyocytes, endothelial cells, and vascular smooth muscle cells. The widespread distribution and alternatively spliced isoforms of FoxP1 suggest that they have distinct functions in different cardiac and vascular cell and tissue types.

It is well known that angiogenesis includes two major biological processes, endothelial cell proliferation and migration. To study the specific effect of FoxP1 on endothelial cell during angiogenesis, due to FoxP1 being highly expressed in endothelial cells, we transfected FoxP1 interfering RNA lentivirus into HUVECs to explore the role of FoxP1 in HUVEC angiogenesis. Our results revealed that the number of proliferative cells decreased distinctly after knockdown of FoxP1 in HUVECs. In addition, the migration and tube-forming ability of *Foxp1* knocked-down HUVECs exhibited an obvious decline compared with that in the control group, and the formed tubules were fewer and more irregular. This result suggests that FoxP1 does play an active role in angiogenesis. This is consistent with the findings of Sebastian et al. [18], who reported that FoxP1 knockdown inhibited endothelial cell proliferation, tube formation, and migration.

In the present study, male Sprague‒Dawley rats and RNA interference lentivirus were used to observe the effect of FoxP1 on angiogenesis after MI in adult rats. Like the results of the cell experiment, endothelial cells in the FoxP1 knockdown group were significantly reduced, with almost no proliferation, while in the MI group, not only was the expression of FoxP1 increased, but the proliferative endothelial cells were much higher than those in the sham group. Studies have demonstrated that FoxP1 is crucial for regulating cell proliferation, apoptosis, oxidative stress, fibrosis, angiogenesis, cardiovascular remodeling, and dysfunction. Enhanced endothelial cell-FoxP1 function protects against pathological cardiac remodeling and improves cardiac insufficiency [27]. Loss of FoxP1 results in pathological cardiac remodeling, increased atherosclerotic lesion formation [16], prolonged occlusive thrombosis, severe cardiac defects, and embryonic death. In contrast, the activation of FoxP1 has broad physiological effects [28], including cell growth, hypertrophy, differentiation, angiogenesis, and cardiac development. More importantly, FoxP1 has anti-inflammatory and anti-atherosclerotic effects in the control of coronary thrombosis and myocardial infarction [26]. Consequently, targeting FoxP1 signaling has emerged as an early warning biomarker and a new therapeutic approach for CVD progression. An in-depth understanding of the role of FoxP1 signaling in the cardiovascular system will facilitate the development of effective interventions.

Early revascularization is extremely important for patients with MI, because it minimizes myocardial cell damage, reduces myocardial tissue collagen deposition, reduces ventricular remodeling, and preserves cardiac function as much as possible [7]. Although there are some shortcomings in this study, FoxP1 overexpression experiments were not performed, and it was not validated in myocardial endothelial cell-specific FoxP1 transgenic rats. The specific mechanism by which FoxP1 regulating angiogenesis during myocardial ischemia has not yet been researched. In this study, we observed that the proliferation, migration, and tube-forming abilities of endothelial cells decreased as the FoxP1 gene was knocked down, which proved that FoxP1 promotes endothelial cell angiogenesis. After MI rats were injected with FoxP1 interfering lentivirus, endothelial cells decreased significantly, and collagen deposition increased. Therefore, FoxP1 causes pleiotropic beneficial actions in angiogenesis in the post-MI heart by promoting proliferation and inhibiting apoptosis of endothelial cells, thereby enhancing cardiac function. Furthermore, we found that hypoxia promotes the expression of FoxP1 in MI rats. This was explained from another perspective and provided a theoretical basis and experimental evidence for the application of therapeutic angiogenesis in the repair of infarcted/ischemic myocardium.

## Supplementary information

FoxP1 interfering RNA lentivirus was successfully transfected into human umbilical vein endothelial cells.

suplementary meterial Uncropped WB

## Data Availability

The datasets generated during and/or analyzed during the current study are available from the corresponding author on reasonable request.
