# Supplementary material for: Transcription factor Foxp1 stimulates angiogenesis in adult rats after myocardial infarction
Source: Cell Death Discov. 2022 Sep 10;8:381. doi: 10.1038/s41420-022-01180-5 (PMC9464245; doi:10.1038/s41420-022-01180-5)

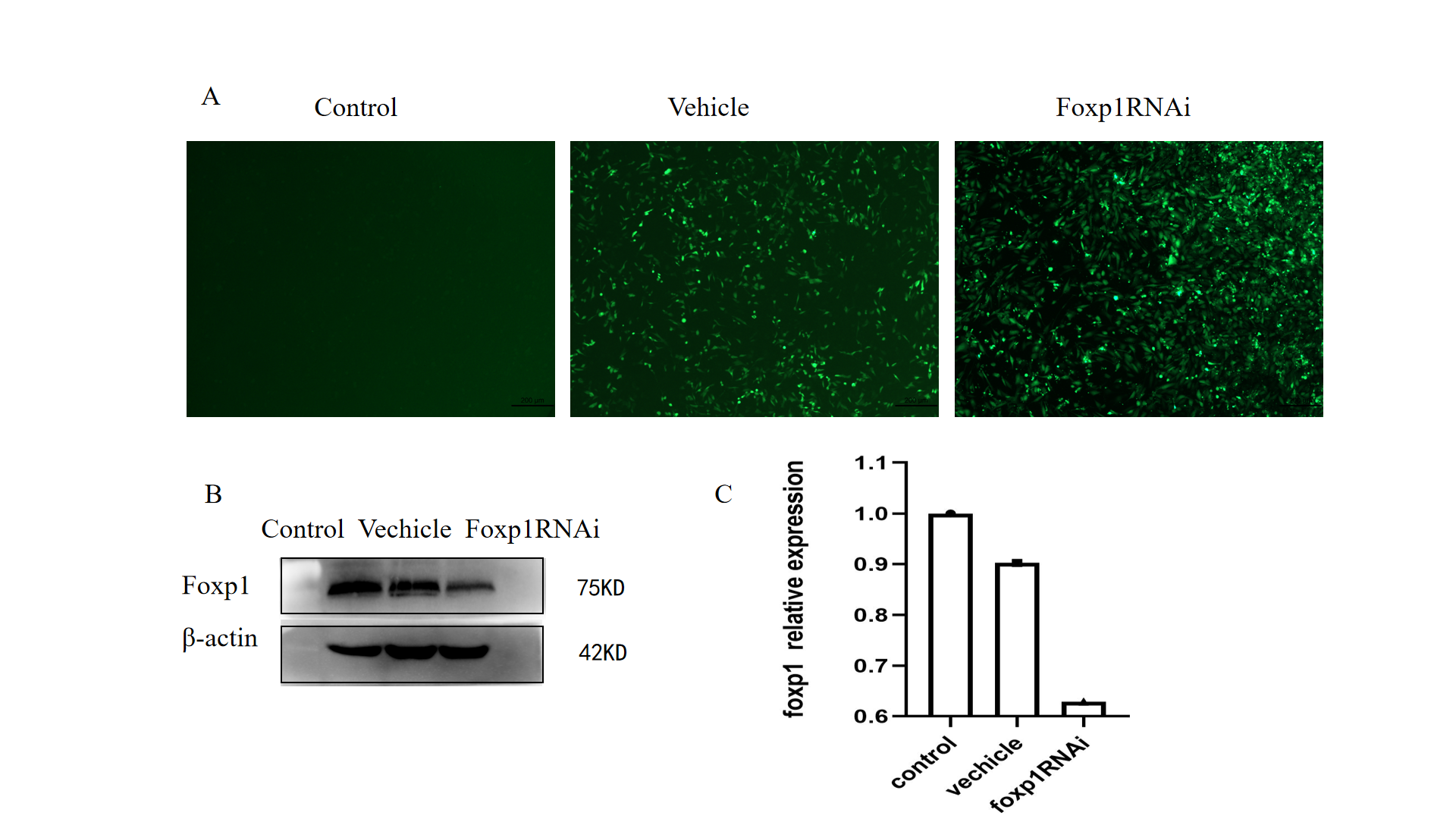


**FigureS1 FoxP1 interfering RNA lentivirus was successfully transfected into human umbilical vein endothelial cells.** A:Compared with the control group, the blank group and the experimental group obviously expressed GFP green fluorescence under the immunofluorescence microscope.B-C:The Western blot experiment of total cell protein showed that the expression of FoxP1 in the experimental group was significantly decreased, which further indicated that FoxP1 interfering lentivirus had been successfully transfected into HUVEC cells.


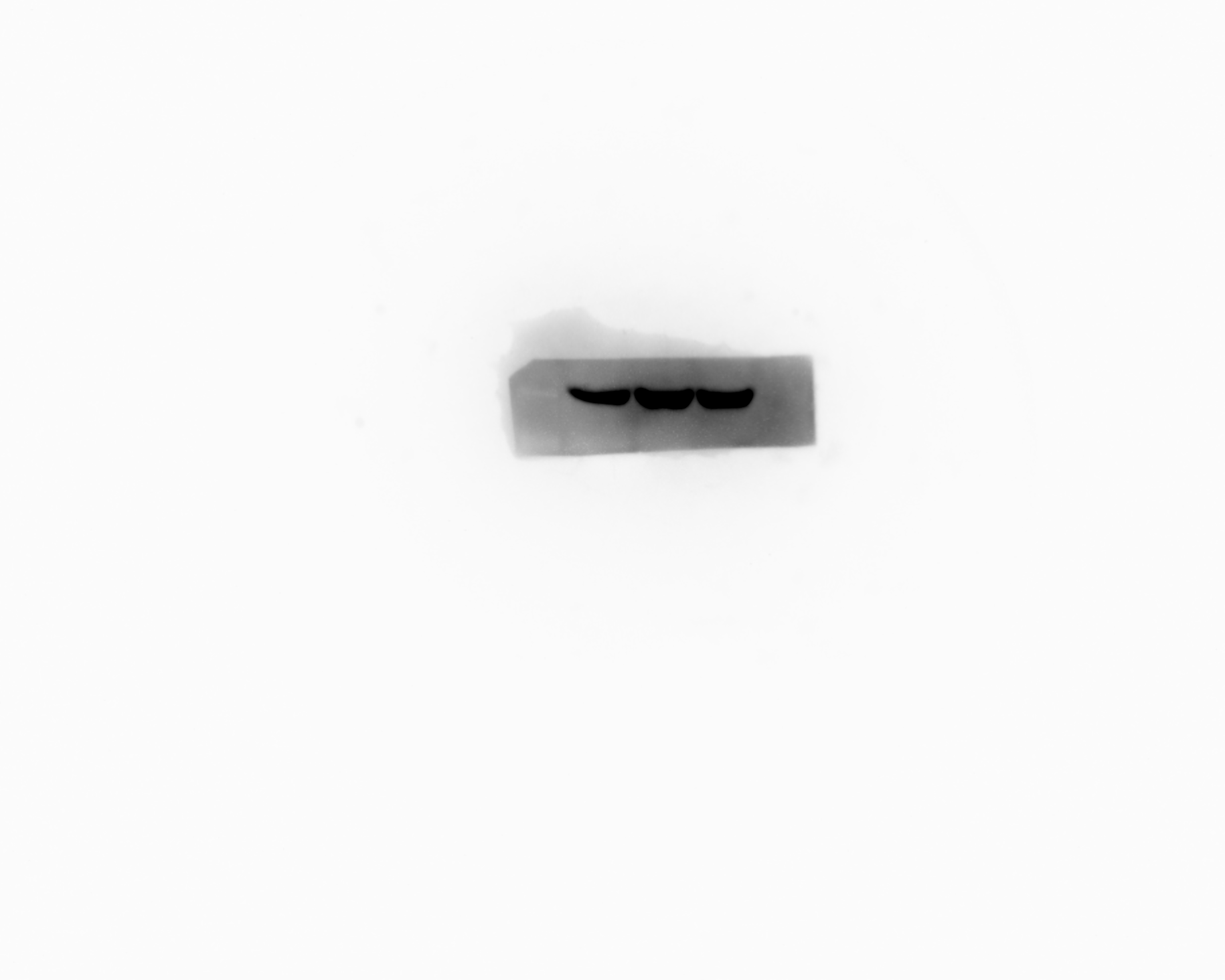

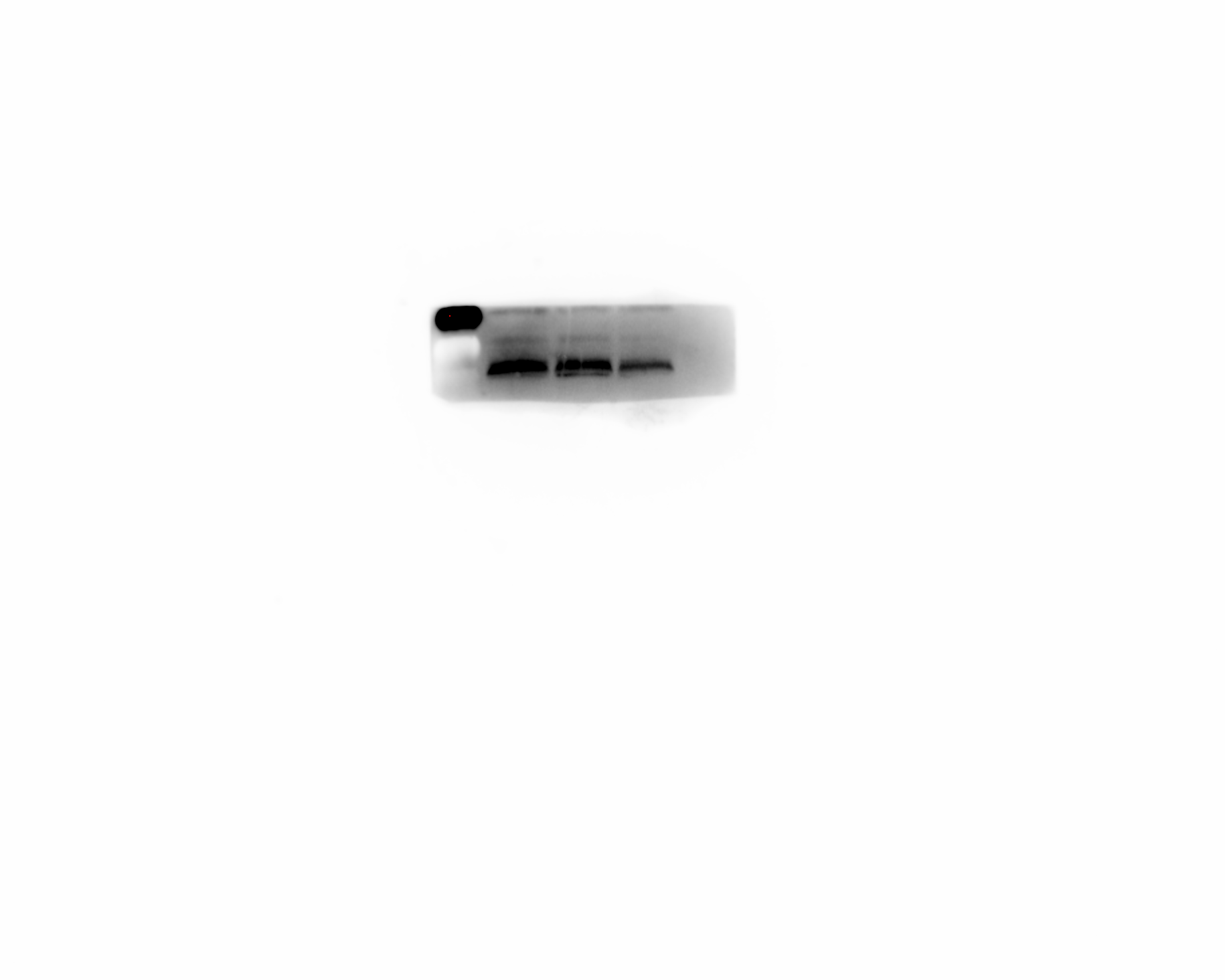

Supplement: Supplementary file 1 — FoxP1 interfering RNA lentivirus was successfully transfected into human umbilical vein endothelial cells. [file 41420_2022_1180_MOESM1_ESM.docx]
