# Supplementary material for: Transcription factor Foxp1 stimulates angiogenesis in adult rats after myocardial infarction
Source: Cell Death Discov. 2022 Sep 10;8:381. doi: 10.1038/s41420-022-01180-5 (PMC9464245; doi:10.1038/s41420-022-01180-5)
Supplement: Supplementary file 2 — suplementary meterial Uncropped WB [file 41420_2022_1180_MOESM2_ESM.docx]

Full and uncropped western blot for Figure 3C


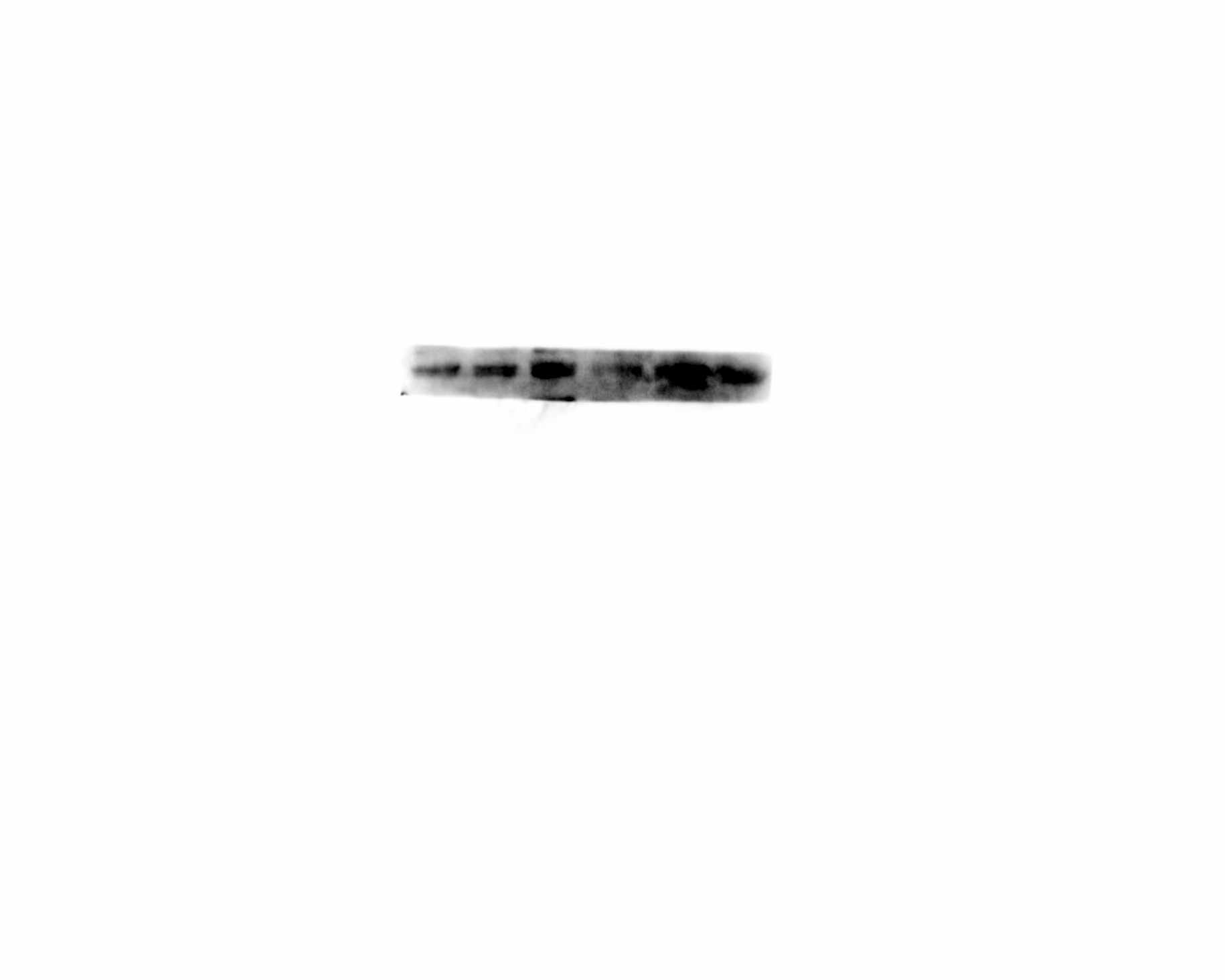


Hypoxia 0h 1h 2h 4h 6h 12hin

Foxp1actin


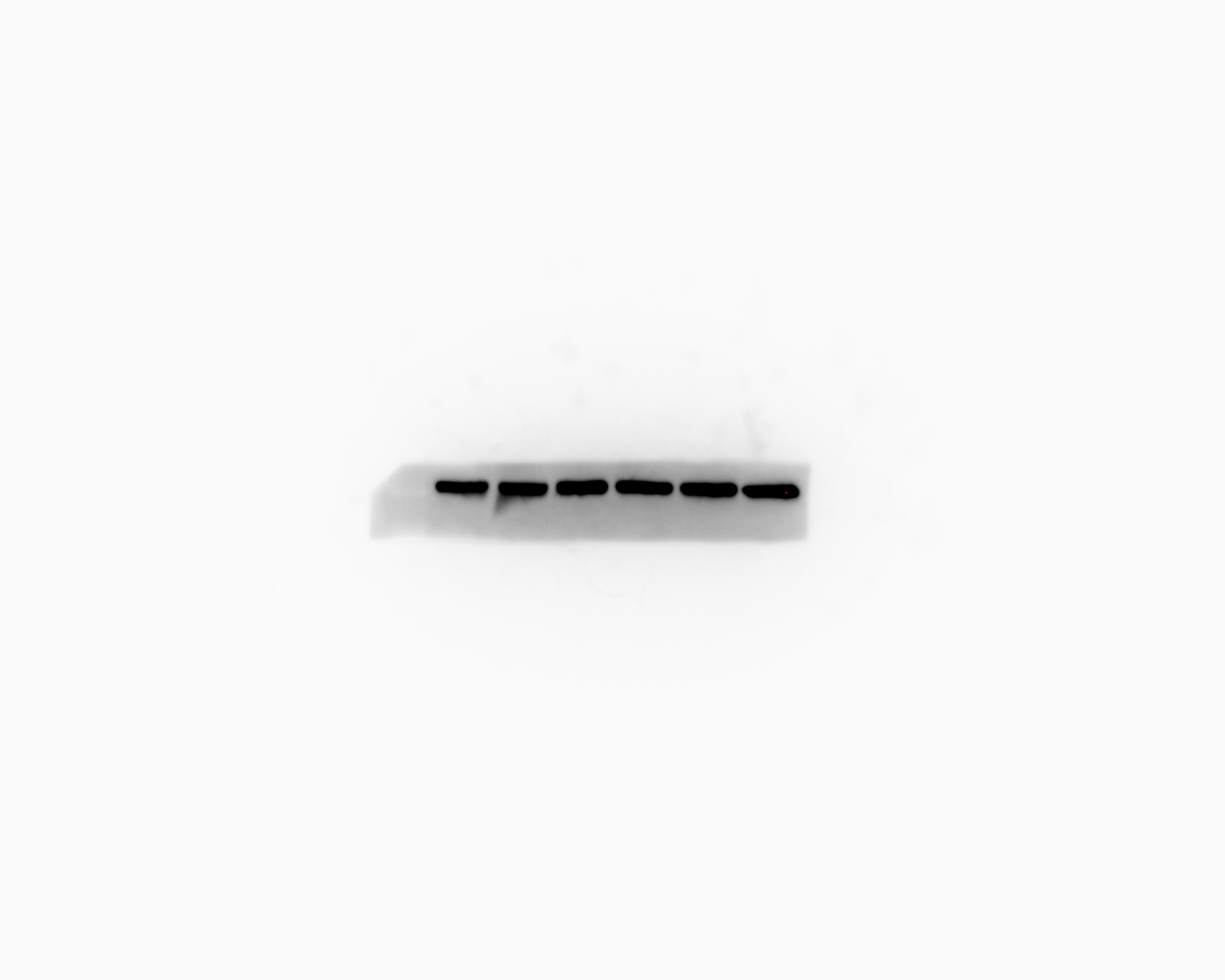

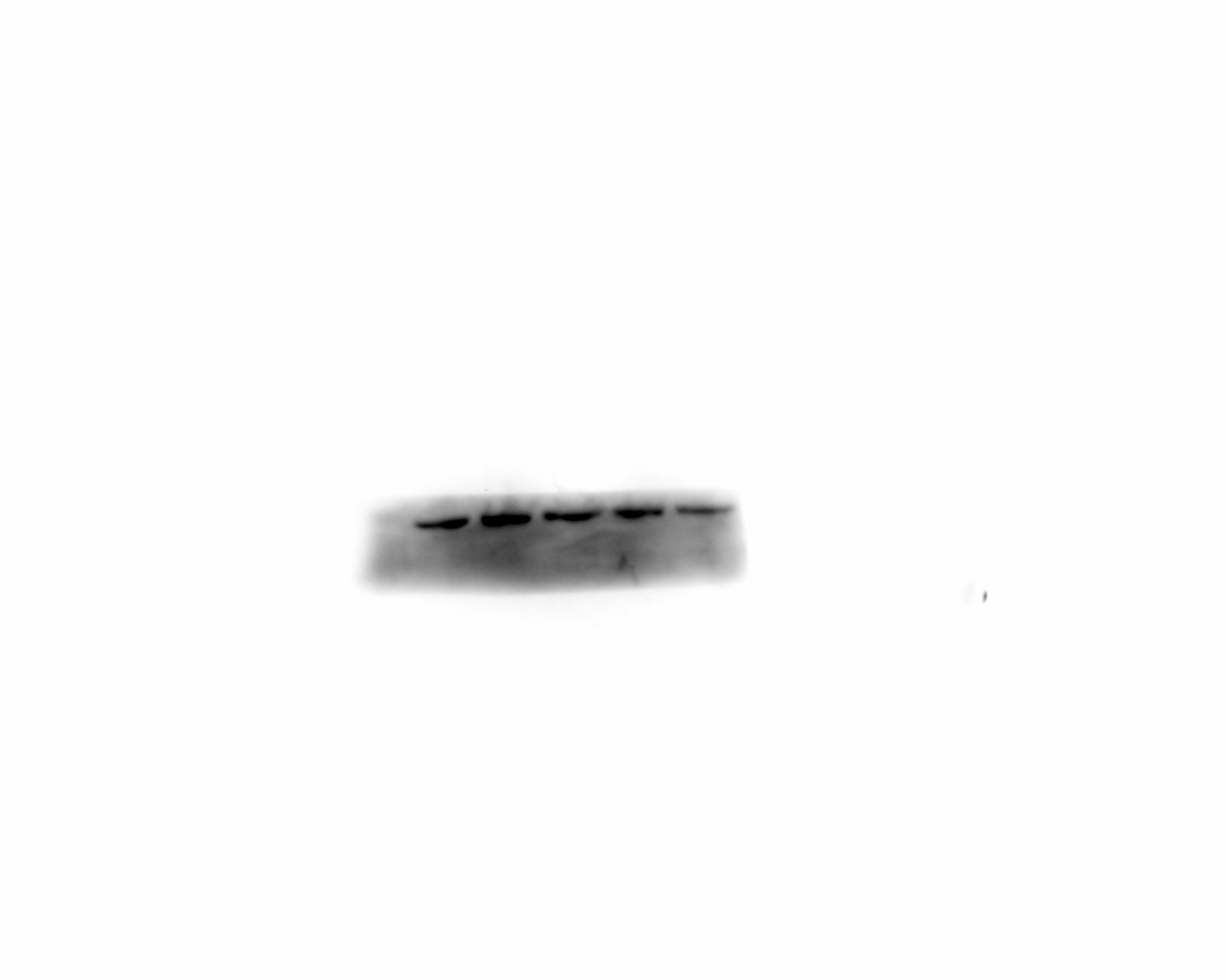


actinactin

Full and uncropped western blot for Figure 3D


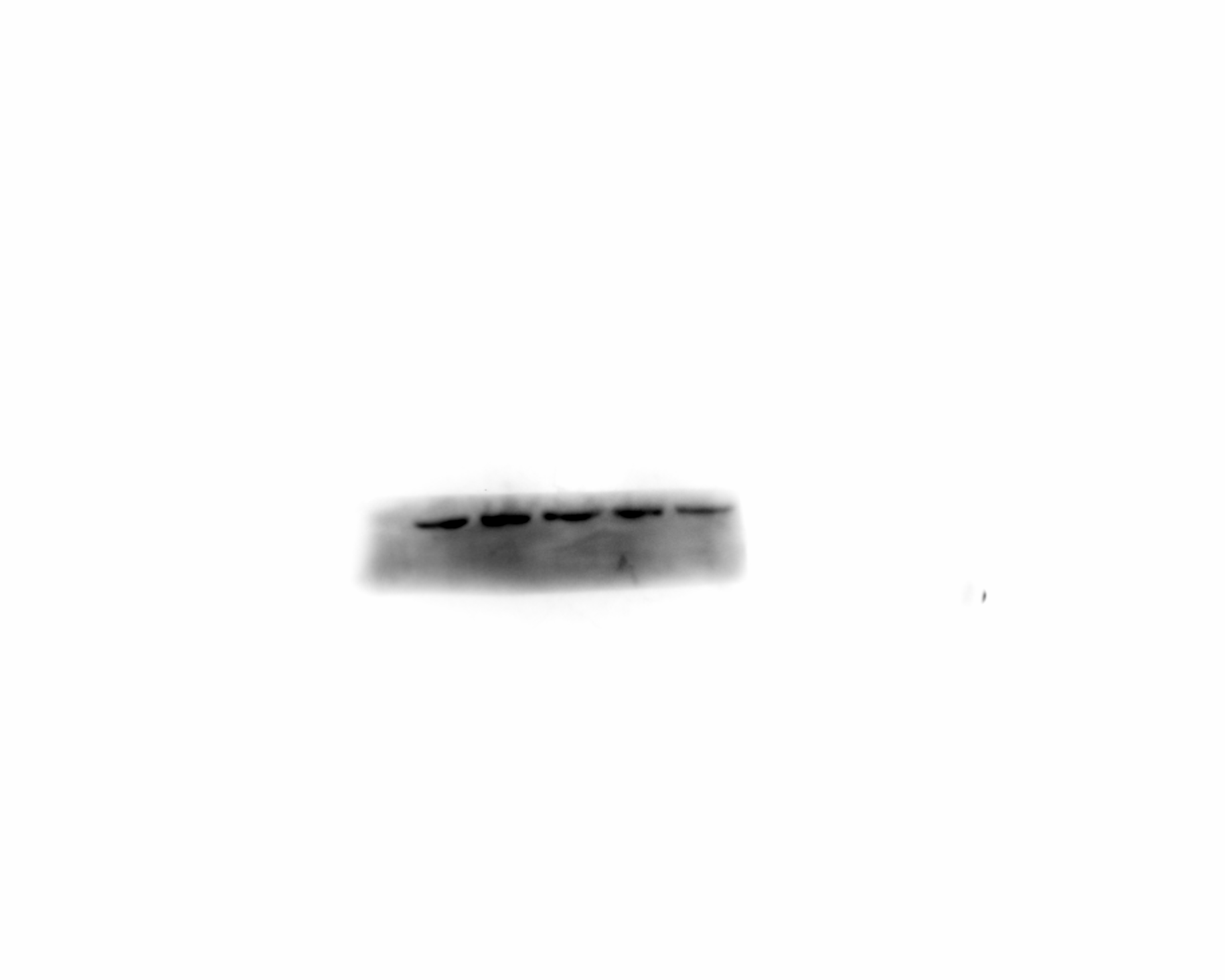


Hypoxia 0h 12h 24h 48h 72h

Foxp1actin


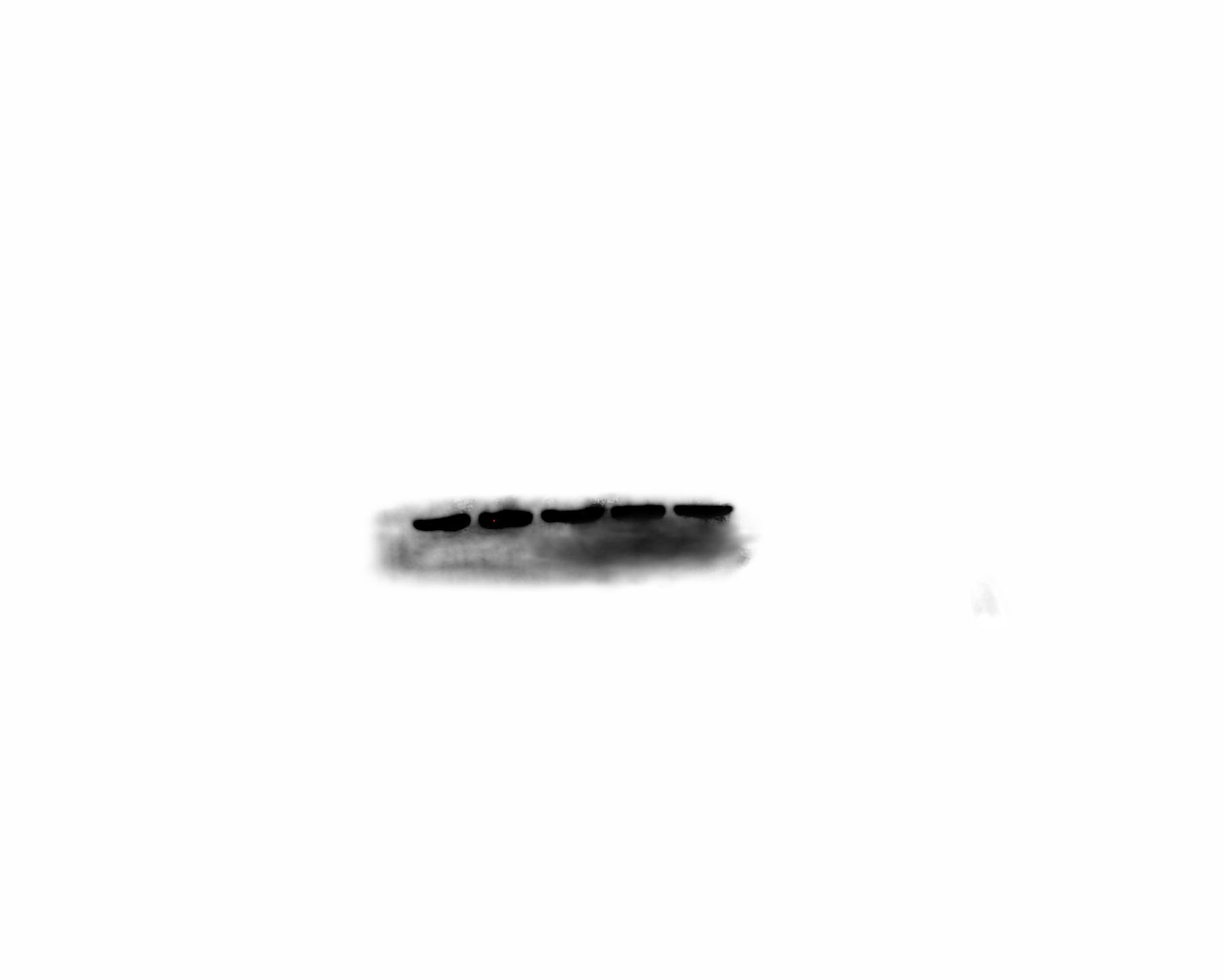


actinactin

Full and uncropped western blot for Figure S1 B


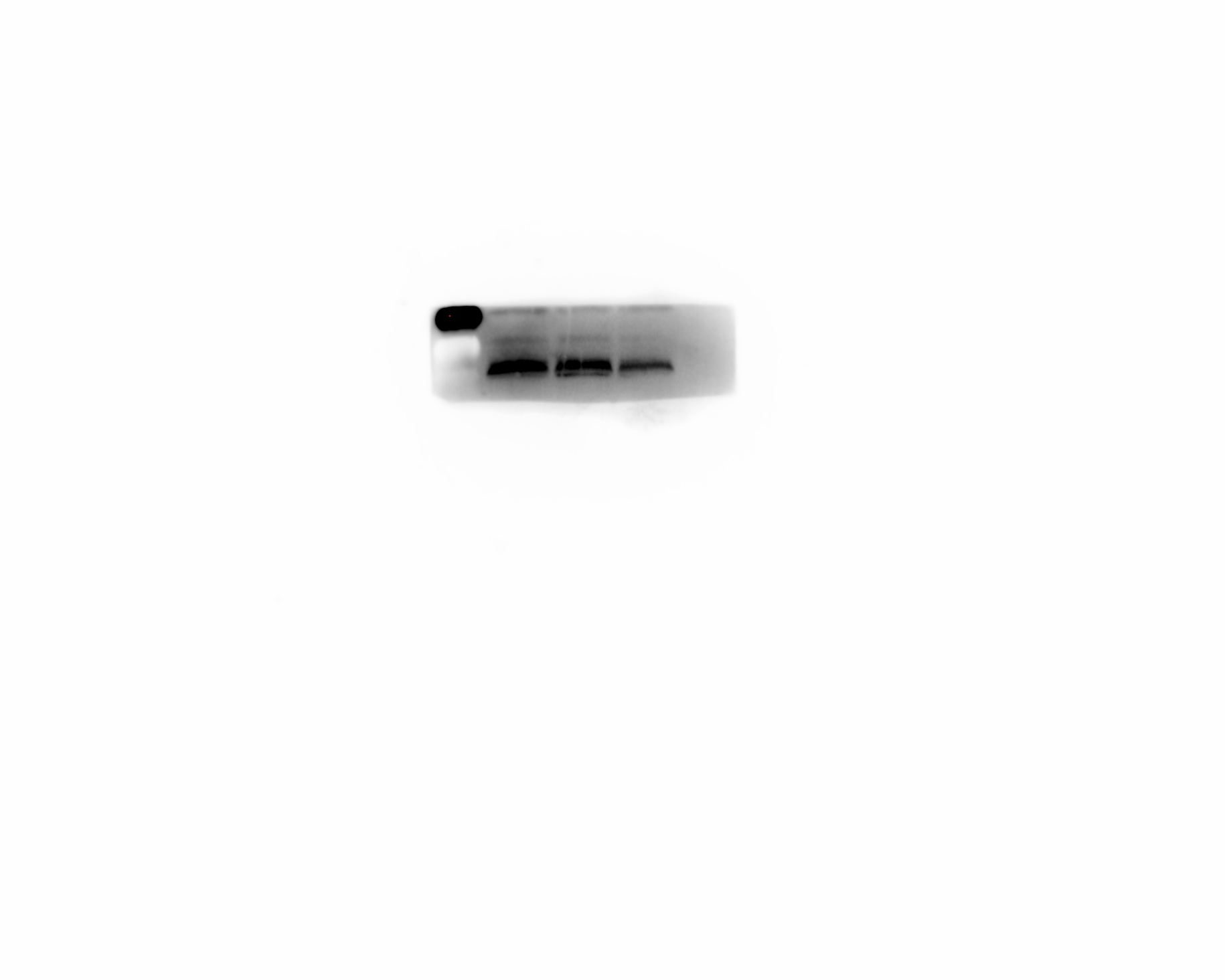


Control Vehicle Foxp1RNAiactin

Foxp1actin


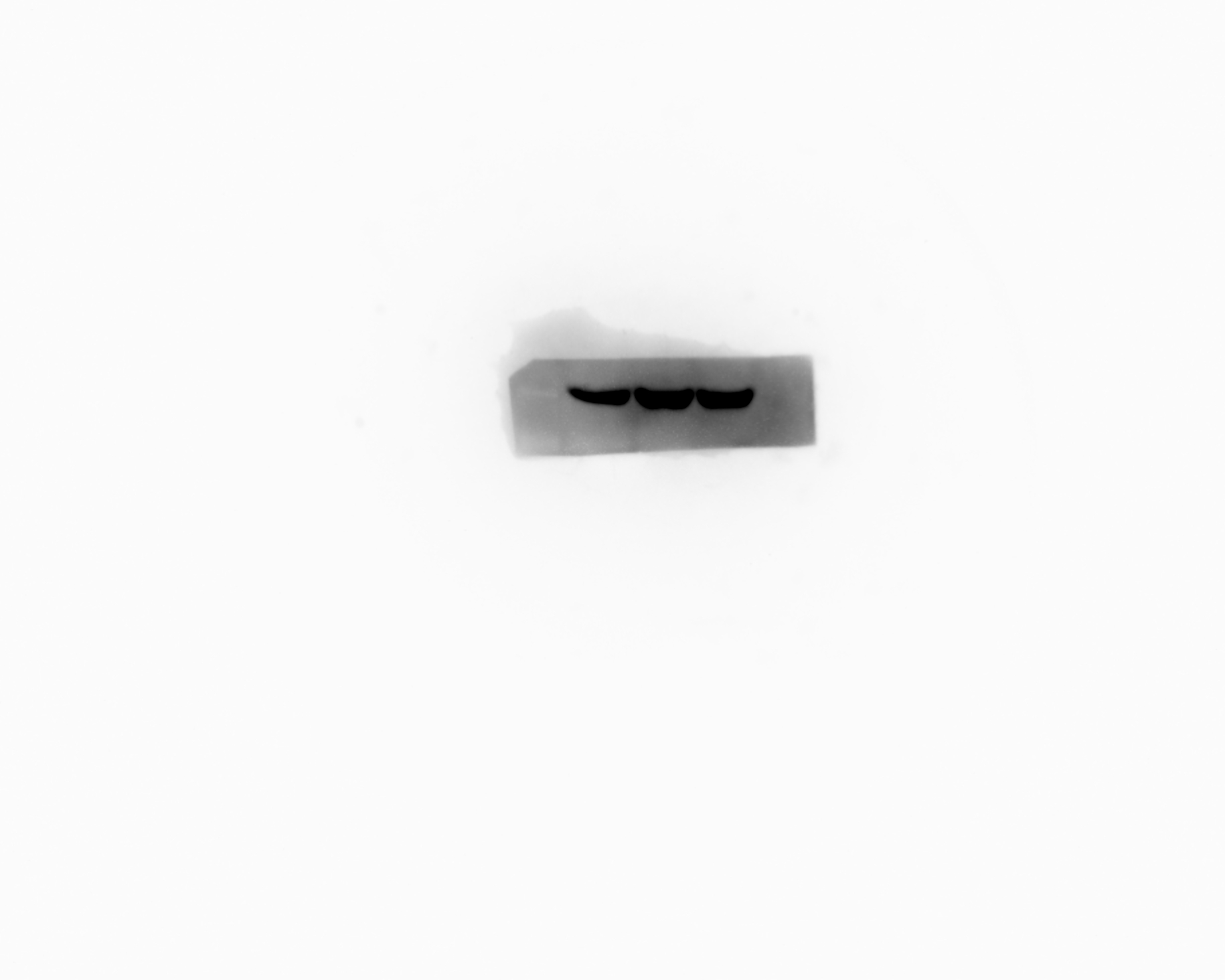


actinactin
